# Case Report: Severe acute respiratory distress by tracheal obstruction due to a congenital thyroid teratoma.

**DOI:** 10.12688/f1000research.6589.2

**Published:** 2015-07-23

**Authors:** Jose Colleti Junior, Uenis Tannuri, Felipe Monti Lora, Eliana Carla Armelin Benites, Walter Koga, Janete Honda Imamura, Patricia Rute Moutinho, Werther Brunow de Carvalho

**Affiliations:** 1 Pediatric Intensive Care Unit, Santa Catarina Hospital, São Paulo, 01310-000, Brazil; 2 Pediatric Surgery Group, Santa Catarina Hospital, São Paulo, 01310-000, Brazil; 3 Pediatric Endocrinology Group, Santa Catarina Hospital, São Paulo, 01310-000, Brazil; 4 Pediatric Oncology Group, Santa Catarina Hospital, São Paulo, 01310-000, Brazil

**Keywords:** thyroid teratoma, pediatric neck mass, pediatric respiratory distress, hypothyroidism, children case report

## Abstract

Congenital teratoma is a rare condition and is a germ cell tumor composed of elements from one or more of the embryonic germ layers and contain tissues usually foreign to the anatomic site of origin. We report a case of a neck tumor diagnosed during pregnancy, initially thought to be a goiter. After birth the neck mass kept growing until it compressed the trachea and produced respiratory failure. The infant had a difficult tracheal intubation because of the compressing mass. The staff decided to surgically remove the neck mass. After that, the infant became eupneic. The histological analysis showed a mature teratoma with no atypias.

## Introduction

Congenital thyroid teratoma is a rare condition ^1, 2^. We report a case of an infant with a neck mass diagnosed by ultrasound during pregnancy which was initially supposed to be a congenital goiter. Two doses of levothyroxine were administered into the amniotic fluid. The goiter kept growing after birth until it caused severe respiratory distress by compressing the trachea, necessitating immediate tracheal intubation. The tumor was surgically resected and the patient went eupneic for the first time in his life. The histological analysis demonstrated a mature teratoma with no atypias. Thyroid hormone substitute therapy was started and the infant is thriving well.

## Case report

A 2-months-and-20-days-old Brazilian white male infant weighing 4.2 kg was admitted to the pediatric intensive care unit of our hospital (Santa Catarina Hospital, São Paulo, Brazil) in acute respiratory distress and was immediately intubated and placed in mechanical ventilation.

**Figure 1.**
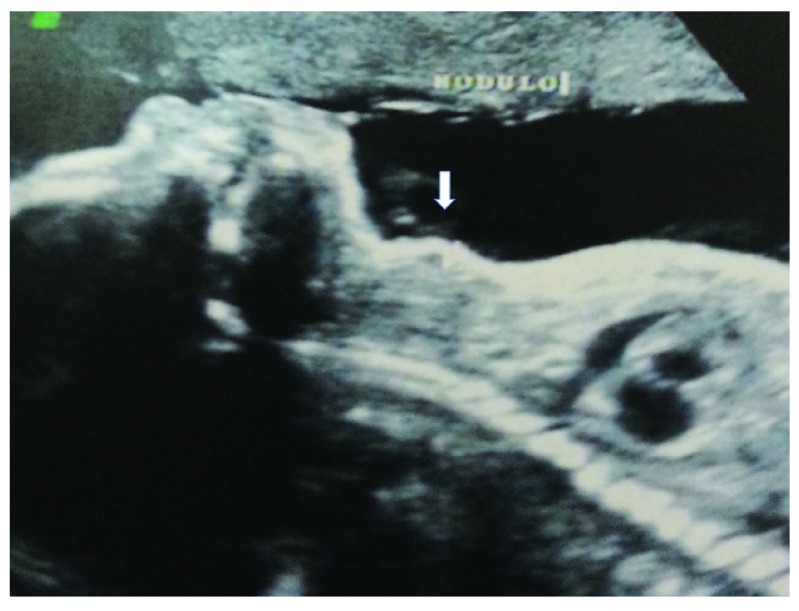
Prenatal ultrasound showing the neck mass.

From a routine ultrasound during pregnancy, the fetus had been diagnosed with a cervical mass, considered initially to be a goiter ( Figure 1) by doctors at another institution. Family history of the mother uncovered a cousin with hypothyroidism. The mother was previously healthy, but after diagnosis of the cervical mass of the fetus, she was tested for thyroid hormones and had hypothyroidism diagnosed during pregnancy (TSH: 5.0 mUI/mL – normal: 0.2 to 3.0 mUI/mL; free T4: 0.7 ng/dL – normal: 0. To 1.3 ng/dL; antithyroglobulin antibodies: 65 U/mL – normal: inferior to 60 U/mL and thyroid antiperoxidase antibodies: 166 UI/mL – normal: inferior to 9 UI/mL). Two single doses of 200µg of levothyroxine were administered into the amniotic fluid, one during the 28th and one during the 31st week of pregnancy, in order to treat the supposed fetal thyroid hormone deficiency. Chorioamnionitis appeared after the second levothyroxine administration which triggered a premature cesarean birth which was undertaken in the other hospital. The premature newborn had sepsis due to maternal infection (chorioamnionitis) and remained in mechanical ventilation for 10 days. After tracheal extubation, he remained in nasal continuous positive airway pressure (CPAPn) for 7 more days, and after that was kept on oxygen therapy for 10 days. He was discharged from the hospital 50 days after birth, still presenting with a laryngeal stridor that was attributed to tracheal malacia by the doctors that initially treated the patient.

After hospital discharge, he was observed by a pediatric endocrinologist who started research on thyroid disorders. Meanwhile, the infant maintained a euthyroid state, receiving no treatment, waiting for more investigation on the cause of the neck mass. However, the cervical mass kept visibly growing, was palpable and the infant presented a laryngeal stridor that was still attributed, by the pediatrician who followed the infant, to laryngomalacia. In the few days preceding hospitalization at our institution, the infant became increasingly dispneic each day, as related by his mother. One day, after choking and vomiting during breastfeeding he became hypotonic and went into acute respiratory distress.

**Figure 2.**
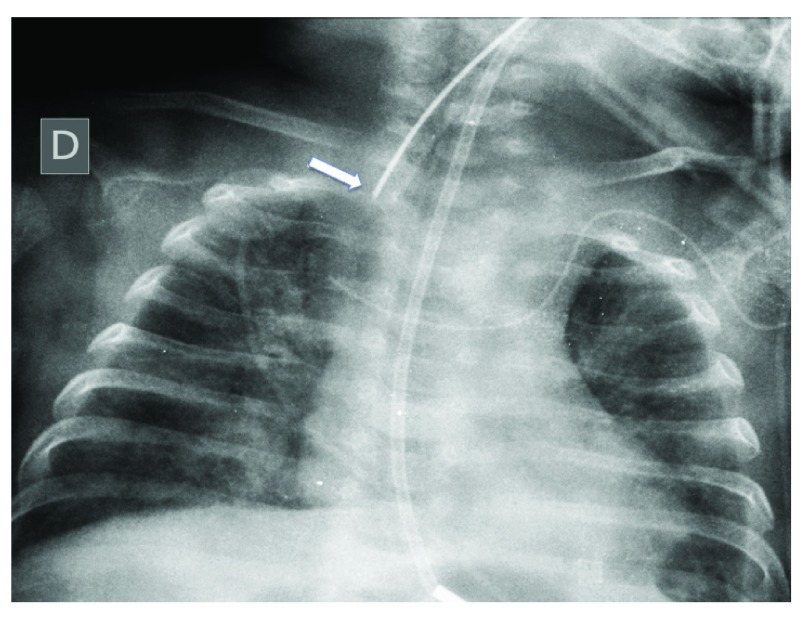
Endotracheal tube displaced to the right position.

**Figure 3.**
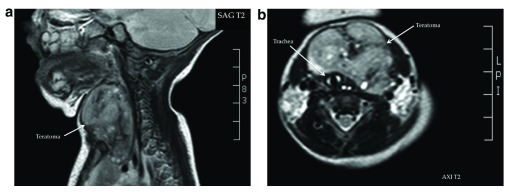
**a**. Sagittal MRI (T2) of the neck showing the teratoma. **b**. Axial MRI (T2) of the neck showing the teratoma and the tracheal displacement.

He was admitted to our pediatric intensive care unit 25 days after he had been discharged from the other hospital, and was immediately intubated. An X-ray showed a small amount of interstitial infiltrate, compatible with aspiration pneumonia. However, the respiratory distress was attributed mainly to an upper airway obstruction. It was difficult to tracheally intubate the infant; only an uncuffed 2.5 mm endotracheal tube (ETT) was able to be inserted into the trachea and it was difficult to place this in the right position. The X-ray after intubation showed the ETT in a high position and the trachea displaced to the right ( Figure 2). Magnetic resonance imaging (MRI) revealed the extent of the cervical mass and its compression on the trachea, and the latter’s subsequent displacement ( Figure 3a and 3b).

Meanwhile, we started investigation into the cause of the neck mass and performed blood tests on the infant: thyroid hormones were in the normal range (free thyroxine (T4): 1.3 ng/dL and thyroid-stimulating hormone (TSH): 4.7 ng/dL). Calcitonin levels, for investigations into potential malignance, were normal (calcitonin: 21 pg/mL), as was the alpha-fetoprotein: 505 µg/L.

**Figure 4.**
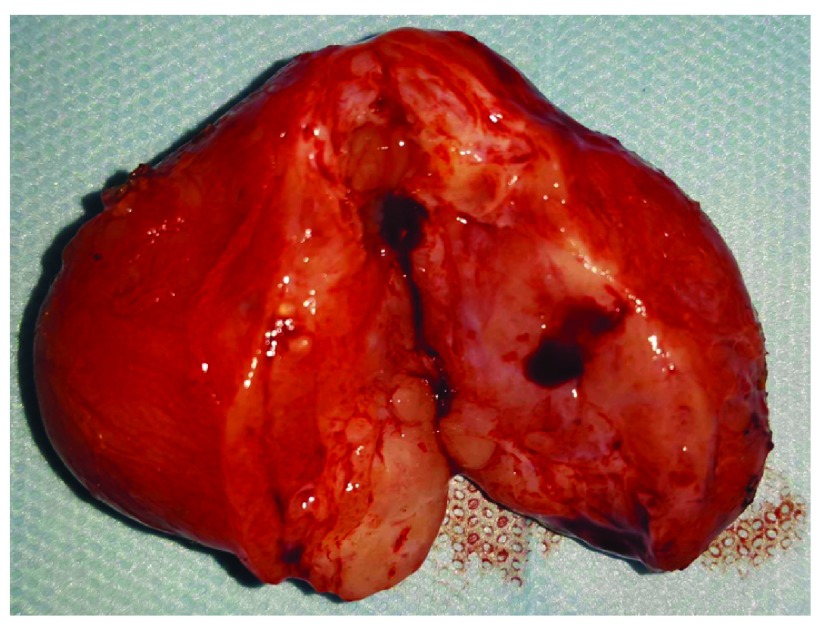
The resected benign teratoma.

We decided to remove the cervical mass, since it was causing the tracheal obstruction. The surgery lasted 35 minutes and was uneventful. The mass was well circumscribed and could be easily dissected, weighed 20 grams and measured 33×61×45 mm Figure 4. The infant returned from surgery in good condition. A bronchoscopy was performed the next day after surgery, during tracheal extubation, which revealed no malacia or any other disorders on the trachea or the upper respiratory tract. The patient has been eupneic since then. The histological analysis revealed a mature teratoma with no atypias or signs of malignancy.

Levothyroxine was started (25µg, once a day) as thyroid hormone substitute therapy and the infant is thriving well according to the pediatric endocrinologist that continues following the patient.

## Discussion

Teratomas originate from multipotent primitive germ cells and result in different tissues, diverging from the anatomical site of origin ^2, 3^. They are most common during early childhood and the most common location is the sacrococcygeal region in children and the gonadal region in adults ^2– 4^. The frequency of these embryonic tumours is about 1:20,000–40,000 live births. However, only 1.5% to 5.5% of all pediatric teratomas are placed in the neck region. These tumours are usually solitary, with no other associated congenital malformations or chromosomal abnormalities ^4^. Although 95% of all teratomas are benign, the cervical teratomas if not properly treated, lead to death in 80% of the cases due to obstructive respiratory distress ^3– 5^.

In this case, the acute clinical presentation of the neck mass with severe respiratory distress, needing ready intervention and immediate tracheal intubation should alert all pediatricians to the risk of these neck masses, and consider it as a potentially fatal case. In the presented case, surgical removal of the neck mass was both diagnostic and therapeutic.

## Conclusion

Thyroid teratoma is rare in infants, it is usually benign, and can cause airway compression depending on the site and size of the mass. The likelihood of a malignant thyroid teratoma is low in infants, however it could be fatal by causing upper airway obstruction. Therefore, surgical resection is required both for diagnosis and treatment. If the surgical removal is a success, the long-term outcome and quality of life should be good ^5^.

## Consent

Written informed consent was obtained from parents of the patient for publication of this case report and any accompanying images. A copy of the written consent is available for review by the editor of this journal.
