# Supplementary material for: Case Report: Severe acute respiratory distress by tracheal obstruction due to a congenital thyroid teratoma
Source: F1000Res. 2015 Jul 23;4:159. Originally published 2015 Jun 22. [Version 2] doi: 10.12688/f1000research.6589.2 (PMC4654446; doi:10.12688/f1000research.6589.2)
Supplement: Peer review history

# Peer review: Referee response for version 2

**DOI:** 10.5256/f1000research.7320.r10593

**Published:** 2015-09-30

**Authors:** Daniel Garros

**Affiliations:** 1 Pediatric Intensive Care Unit, Stollery Children’s Hospital, Edmonton, AB, Canada

The authors describe an interesting case, with a positive outcome despite some delay in diagnosis.

The title and the description of the case are all well written.

However, the **discussion and the conclusion** require more work. This case brings up an important and not so rare clinical scenario in Paediatric Critical Care, i.e. the difficulties inherent to extrinsic airway compression by a tumour. The authors, on their discussion, should take advantage of this case and discuss the approach to airway extrinsic compression, the use of extra-corporeal life support (ECLS) as a back-up plan, who should be present in such situations (ENT, anaesthesia), etc… The clinician could barely secure the child’s airway, and the child survived and was able to have the teratoma resected. But it could have been a disastrous outcome. There are protocols for such scenarios, and they should be mentioned and described for the reader to be prepared when facing with similar situations

I have read this submission. I believe that I have an appropriate level of expertise to confirm that it is of an acceptable scientific standard, however I have significant reservations, as outlined above.

# Peer review: Referee response for version 2

**DOI:** 10.5256/f1000research.7320.r9516

**Published:** 2015-08-25

**Authors:** Francisco Eulógio Martinez, Walusa Assad Gonçalves-Ferri

**Affiliations:** 1 Department of Pediatrics, Faculty of Medicine of Ribeirão Preto, University of São Paulo, Ribeirão Preto, Brazil

Coletti *et al *described in a case report of severe acute respiratory distress by tracheal obstruction due to congenital thyroid teratoma. This case report adds knowledge to the literature, not only by tumor description, but also the clinical management of the case.

Tumor description is interesting, however I believe that the authors should also discuss in this article the clinical management of malacia in premature, since this is very common in neonatal clinic. I believe that this discussion would make the most interesting article and is also an opportunity to develop the discussion of the article, which I believe is short. For this reason there are some points which need to be considered and are necessary clarifications

Add data:

1. The patient was born with what gestational age?
2. After birth was not performed control with imaging for the mass?
3. What are the results of thyroid hormones tests after birth?
4. The staff investigated other causes for laryngeal stridor before discharge?

Suggestions:

The discussion could  discuss the differential diagnosis of stridor. The staff not investigated a patient with stridor, attributing the malacia. Many patients in neonatology have stridor and I believe that this article could show that there is need for investigation in stridor, because sometimes the differential diagnosis can be serious pathologies.

Case reports are interesting, but I believe it should have a good discussion of the various aspects involved in the pathology, in which case I would like that you develop the discussion and mainly develop the discussion about the differential diagnosis.

We have read this submission. We believe that we have an appropriate level of expertise to confirm that it is of an acceptable scientific standard.

# Response

**Published:** 2015-09-10

**Authors:** Jose Colleti Junior

**Affiliations:** Hospital Santa Catarina, Brazil

I would like to thank Dr Martinez and colleague for the remarks and suggestions above.

Answering the questions regarding this case report:

1. The patient was born with 31 weeks of gestational age, immediately after the second administration of levothyroxine in the amniotic fluid due to a chorioamnionitis.
2. After birth new imaging control was done in the previous hospital and in our medical center (figure 3).
3. After first hospital discharge, the patient was followed by a pediatric endocrinologist who asked for thyroid hormone tests which resulted normal for the age (TSH=1.8 mUI/mL – normal: 0.8 to 6.0 mUI/mL; free T4: 0.9 ng/dL – normal= 0.7 to 1.5 ng/dL).
4. Since the patient was born in other hospital we could not know if the staff did any other investigation for other causes of laryngeal stridor. In our medical center, the patient was admitted in a critical clinical status and the surgery was promptly performed.

I hope I have clarified your doubts and concerns about this case report. Thank you for the review.
